# Gaze in Visual Search Is Guided More Efficiently by Positive Cues than by Negative Cues

**DOI:** 10.1371/journal.pone.0145910

**Published:** 2015-12-30

**Authors:** Günter Kugler, Bernard Marius ‘t Hart, Stefan Kohlbecher, Wolfgang Einhäuser, Erich Schneider

**Affiliations:** 1 Institute of Clinical Neurosciences, University of Munich, Munich, Germany; 2 German Center for Vertigo and Balance Disorders, University of Munich, Munich, Germany; 3 Neurophysics, Philipps University Marburg, Marburg, Germany; 4 Centre for Vision Research, York University, Toronto, Ontario, Canada; 5 Institute of Physics, Chemnitz University of Technology, Chemnitz, Germany; 6 Institute of Medical Technology, Brandenburg University of Technology Cottbus–Senftenberg, Senftenberg, Germany; University of Verona, ITALY

## Abstract

Visual search can be accelerated when properties of the target are known. Such knowledge allows the searcher to direct attention to items sharing these properties. Recent work indicates that information about properties of non-targets (i.e., negative cues) can also guide search. In the present study, we examine whether negative cues lead to different search behavior compared to positive cues. We asked observers to search for a target defined by a certain shape singleton (broken line among solid lines). Each line was embedded in a colored disk. In “positive cue” blocks, participants were informed about possible colors of the target item. In “negative cue” blocks, the participants were informed about colors that could not contain the target. Search displays were designed such that with both the positive and negative cues, the same number of items could potentially contain the broken line (“relevant items”). Thus, both cues were equally informative. We measured response times and eye movements. Participants exhibited longer response times when provided with negative cues compared to positive cues. Although negative cues did guide the eyes to relevant items, there were marked differences in eye movements. Negative cues resulted in smaller proportions of fixations on relevant items, longer duration of fixations and in higher rates of fixations per item as compared to positive cues. The effectiveness of both cue types, as measured by fixations on relevant items, increased over the course of each search. In sum, a negative color cue can guide attention to relevant items, but it is less efficient than a positive cue of the same informational value.

## Introduction

Visual search is one of the most widely used paradigms to study human visual attention [1] [2] [3] [4]. Performing a visual search is thought to be based on two mechanisms [5] [6]: (1) A pre-attentive *parallel* mechanism processes the whole scene. This explains why some search tasks can be performed “efficiently”, in that, the duration of the search is independent of the number of items. Such “pop-out” typically occurs when a target distinguishes itself from non-targets (distractors) by the presence of an elementary feature. Typical examples include the search for a singular color, such as a red dot among green ones, or a unique orientation, such as a vertical bar among horizontal bars. Also, absent features (e.g., an O amongst Qs) are often more difficult to find than present features (e.g., a Q amongst Os). (2) An attentive *serial* mechanism processes one item after another, if the parallel process is not able to distinguish the target from non-targets (distractors or foils). In this case, the time needed to find the target increases with the number of items present (e.g. searching for a “T” among “L”s). Parallel and serial search are likely only extremes on a continuum from efficient to inefficient search [7].

Aside from efficient processing of the properties of the visual scene, search can benefit from prior knowledge about properties of the target. This can be achieved by cueing the likely location of an item on which to perform a discrimination task [8]. Alternatively, features of target items can be cued. Cues about the targets’ color, size, or shape lead to shorter search times in that a larger proportion of fixations will fall on items that share the cued property with the target [9]. Such behavior is modeled by “Guided Search” (GS) [2] [9] [10], which is one among several other visual search models which assume that items or locations that share a feature value with the target are preferentially processed. Other such models are for example Feature Integration Theory [1], FeatureGate [11], or Attentional Engagement Theory [3]; for reviews, see [12] [13]. These models combine the parallel and serial mechanism.

Accordingly, search is modeled as a serial inspection of items where the focus of attention is “guided” by the parallel evaluation of features of the scene. The parallel evaluation of the search array provides a decision whether items in the periphery should be included into the attentive (i.e., serial) part of search. Thus, when searching for a red “T” among green and red “L”s, serial search can be directed to the red items. This way, a cue about target features facilitates search.

Models of visual search assume that the inclusion of items in the serial progression of attention is based on the *presence* of certain feature values. Thereby they neglect that knowledge about the *absence* of certain feature values in target item or location may also facilitate search. The concept of negative cues is an extension of–but distinct from–*defining* targets by the absence of a feature altogether [14]. There is evidence that visual search might benefit from negative cues [15] [16] [17] [18] [19], and that there are costs if potential distractors are absent [20], but it is unclear how gaze behavior might be influenced by this type of cueing. Here, we investigate the impact of negative cueing on guidance in visual search, by examining the following questions: (1) Is negative cueing more, less, or equally efficient, than positive cueing, and how does it compare to neutral cues? (2) Does negative cueing guide visual search differently and what are the underlying mechanisms? (3) How does the effectiveness of a positive or negative cue develop over the course of a trial? To address these questions, we designed a search paradigm and measured response time, search accuracy and those eye-movement parameters that are considered a good proxy of visual attention [21] [22] in two experiments. In experiment 1, trials were matched such that for each positive-cue trial there was a corresponding negative-cue trial that used the same search display. Both experiments were designed such that negative and positive cues conveyed the same information about possible target locations. In addition, in experiment 2 the number of colors of potential target items were matched between corresponding positive-cue and negative-cue trials.

## Methods

**Fig 1:**
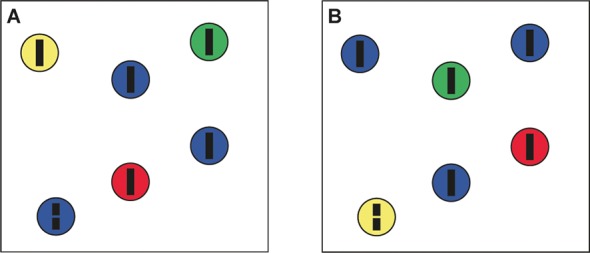
Search array examples. A: Array in positive cue type: “target will be blue”. B: Array in negative cue type: “target will not be blue”. Both arrays have the same spatial configuration of items and the target. The colors of the items were switched. The target is at the same location in the lower left corner. Search arrays are not to scale.

In two experiments, observers were asked to perform a search task among a set of items. All items consisted of a colored disk, in which a vertical black line (0.37 degrees of visual angle) was embedded. In half of the trials, one line had a gap (0.06 degrees) in its center (Fig 1). The item that contained the broken line will be referred to as “target”, the remaining (i.e., non-target) items as “foils”. Identifying the gap required foveation of the target, as verified by pilot measurements, thus encouraging serial inspection of items. Observers were asked to report in each trial whether such a broken line was present or absent as quickly and accurately as possible, by pressing a corresponding button on a modified keypad with two buttons. The display was on until the participant responded. Times from onset of the search array until button activations were recorded (“response time”).

A set of 8 colors equal in luminance (6.3±0.05 cd/m^2^) was chosen to be easy to differentiate by name and hue (CIE-coordinates: red: x = 0.59, y = 0.33; green: x = 0.29, y = 0.57; yellow: x = 0.41, y = 0.48; blue: x = 0.15, y = 0.07, cyan: x = 0.21, y = 0.28; orange: x = 0.49, y = 0.42; purple: x = 0.20, y = 0.10; pink: x = 0.37, y = 0.21). The CIE coordinates (x,y) and the luminance (Y) of the color set were measured with a photospectrometer (PR-655, Photo Research Inc.). In “positive cue” blocks, participants were informed about the possible color(s) of the disk containing the target. In “negative cue” blocks, the participants were informed about the color(s) of disks that would not contain the target.

### Experiment 1

#### Design of search arrays

Search displays were designed such that for both the positive and the negative cues the same number of items could potentially be the target. Therefore, both cues were equally informative. Half of the items in a search array were cued by color. In the case of a positive cue, the cued color identified the items where the target could be (hereafter: “relevant items”), whereas a negative cue identified the items where the target could not be (hereafter: “irrelevant items”). We used five array set sizes (6, 12, 18, 24 and 30 items) for search. Search arrays were created once with the items at random spatial coordinates with a minimum separation of 3° of visual angle (measured from center to center). First, a set of 80 search arrays for the positive cue condition was created as follows: For each of the 8 colors and five set sizes, one array with a target present and one array with only foils (target absent) was created. This means there were 8 sets of 10 arrays, one set for each of the 8 colors. Half of the items were of this color. This color could appear as cue for the array, to either indicate that those items would contain the target if present (positive cue) or never contained the target (negative cue). The other half of the items were assigned evenly to one of three other colors selected randomly from the remaining set of colors for each array (Fig 1A). Varying the non-cued colors encouraged using the cued colors to reject items for inspection following a negative cue, as otherwise the relevant color would be constant throughout a block and participants might have ignored negative cues after a few trials. Each of the 80 positive cue arrays was matched by a negative cue array with identical spatial configuration but switched colors (Fig 1B). Thus, the total number of search arrays and trials was 160.

#### Procedure

**Fig 2:**
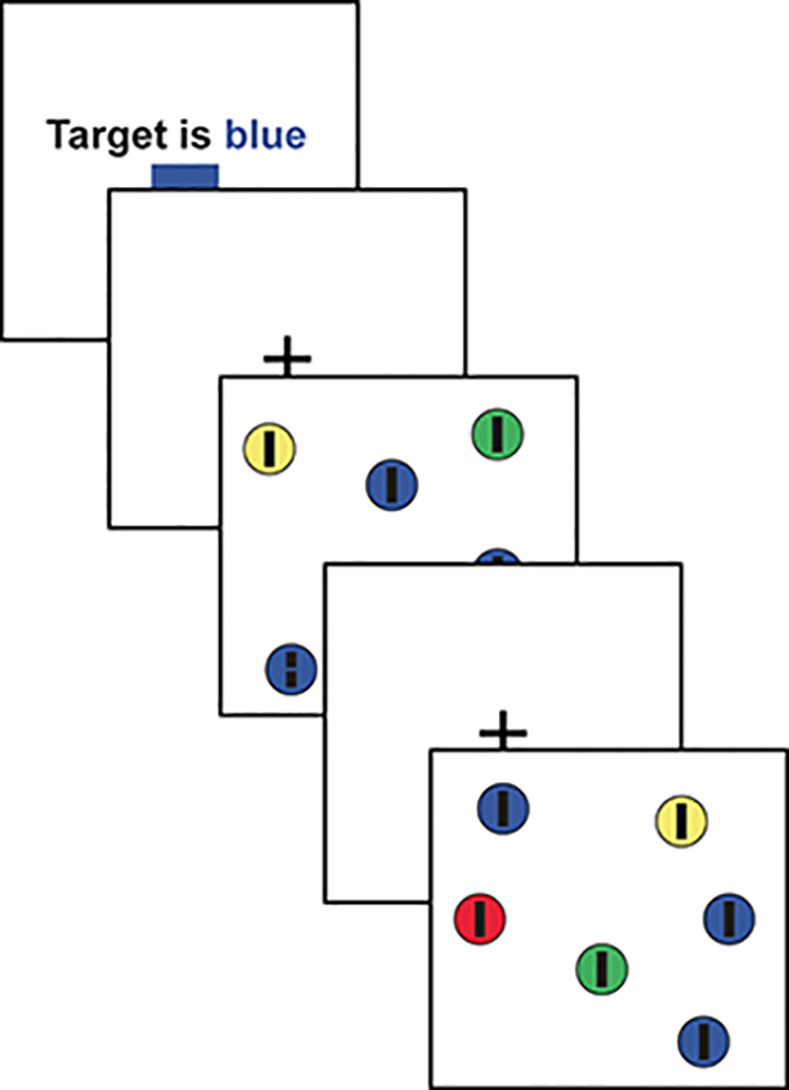
Sequence of search arrays. At the start of each block, the cue is shown. Then, a fixation cross is displayed for 3s, followed by a search array. After 10 search arrays, the block is complete, and a new block begins.

After ten practice trials, sixteen blocks with ten search arrays each were presented in randomized order for each participant. At the start of each block, the participants were given a cue for the whole block (see the sequence in Fig 2). For a block chosen from the positive cue set, the participants were told that if there is a target, it will definitely be of a specified color (“positive cue”). For the corresponding block from the negative cue set (i.e. the same block with switched colors), the participants were told the same color, but cued that if there is a target, it will not be of that color (“negative cue”). These cues were always valid and the participants were informed so. This amounted to a total of 16 cues given.

Each of the colors was used as cued color twice; once for the positive cue and once for the negative cue. Each of the blocks consisted of 5 “target absent” and 5 “target present” search arrays, one for each array size. Before each block, the cue was shown with the given color, which was identified by color name and the actual hue. The participants could start the next block by pressing one of the buttons when they were ready. Before each search array, a fixation cross was presented for 3 seconds. The whole procedure took about 30 minutes, including breaks. After completing the experiment the participants were debriefed and explicitly queried about whether they realized that every spatial distribution was present twice. No participant reported having realized this during the experiment.

#### Participants

In total, 21 subjects (aged 27 ±7 years, mean and standard deviation, 9 female) with normal, or corrected to normal vision participated in the experiment. On a subset of 14 subjects (aged 26 ±7 years, mean and standard deviation, 7 female), eye-tracking data could be obtained. All analyses reported here pertain to these 14 subjects. For reaction time and accuracy data, we performed the same analysis in all 21 observers, and found that exactly the same effects were significant (at a 5% alpha level) as with the reduced dataset. All participants gave informed written consent and were paid compensation for participating. All procedures were in accordance with institutional guidelines and the declaration of Helsinki, and they were approved by the FB04 ethics committee of the Philipps University Marburg.

#### Data analysis

The search accuracy of all participants was determined by evaluating the proportion of correct responses. These were analyzed with a generalized mixed-effects model with a logistic link function, separated for *target presence* (target absent and target present), with the factors *cue type* (positive cue, negative cue) and *array set size* (6, 12, 18, 24, 30). We also calculated the sensitivity index (d’) and criterion (c) separately for positively and negatively cued trials to verify that response criteria were comparable for both cue types. Only trials with correct responses (95.8% of trials in experiment 1) were used for analysis of reaction times and fixations. For every participant the mean response time for all remaining trials with the same array size, cue type, and target presence was calculated. A repeated measures ANOVA (using a general linear model) on response time as the dependent variable was conducted with the same two factors *cue type* and *set size*, again separated by *target presence*. In cases where Mauchly’s test indicated that sphericity was violated, we report Greenhouse-Geisser corrected p-values.

In the analyses of eye movement data, three variables were investigated: the proportion of fixations on relevant items, mean fixation durations, and mean number of fixations per item. The combination of the latter two corresponds to the mean dwell time per fixated item. To calculate these three variables all fixations assigned to an item were used, but only target absent trials were regarded. In target-present trials, any fixations and refixations on the target at the end of search will bias the proportion of fixations on relevant items, as the target is always a relevant item. Since this may obscure insights into the ongoing search process, the target-present trials were left out of these analyses. In addition, the correlations of the time until the target is fixated (if present) with the response time were calculated. For this analysis, only target present trials were regarded. In experiment 1, the fixations were identified with a dispersion-based algorithm [23] (dispersion threshold 0.9°, duration threshold 50 ms). To each fixation, the nearest object on the screen was attributed if its center was closer than 3° of visual angle, thus 89% of fixations were attributed to specific items for experiment 1. To calculate the proportion of fixations on relevant items, the number of fixated relevant items as well as the total number of fixated items were obtained for each participant, array size and cue type. Relevant items were either items of the color specified in the instruction in the positive cue or items of all other colors in the negative cue. A two-way repeated measures ANOVA was calculated with the dependent variable proportion of fixations on relevant items with the factors *cue type* and *array set size*. Statistical analysis was performed with R version 3.0.2. [24]

#### Setup

The experiment was run on a MacPro, Apple Inc., and programmed in Matlab 2010a, Mathworks Inc., using the Psychophysics Toolbox extensions, version 3.0.10 [25] [26] [27]. The search arrays were presented on a color-calibrated and characterized 19 inch CRT screen (EIZO FlexScan F730), with a resolution of 1024x768, a refresh rate of 85 Hz and a luminance range of 1.4 cd/m^2^ (“black”) to 150 cd/m^2^ (“white”) in a room with negligible ambient light. Participants were placed in front of the screen with a chin rest at 65 cm distance to the screen (eyes to screen center). For measurement of the eye movements each participant wore a binocular infrared eye-tracker (“EyeSeeCam” [28]), with a sampling rate of 120 Hz. A third camera was used to track the head’s position and orientation relative to LEDs placed on the borders of the screen. Eye-to-screen calibration was performed with a 20-point calibration protocol. Gaze-on-screen positions were calculated online and recorded.

### Experiment 2

In experiment 1, always one out of four colors was cued, either negatively or positively. Consequently, in a positive-cue search array all potential targets were of one color, while in a negative-cue search array potential targets could be of one of three colors. A strategy that participants may use in this setup is to translate the negative cue into a positive cue: first they would identify the colors that were not cued and then they would search for these three remaining colors. This would imply that participants did not use the “template for rejection”, but created their own “template for inclusion” instead. This strategy would have introduced the need to switch between multiple colors in the negative cue condition of experiment 1. Thus, in experiment 2 we introduced a paradigm where we matched the number of colors the targets could be, in both negative cue and positive cue conditions to account for a possible cost of color-switching. In addition, we included a neutral condition to allow measuring benefits of positive and negative cues in terms of performance as well. Since cues were repeated throughout a block, this might have resulted in more efficient search later in the block [16]. To test if this was systematically different between the cue types, we performed a paired t-test on the average decrease in RT from the first trial in each block to the last, comparing positive and negative cues across all other variables.

#### Design of search arrays

Search arrays consisted of either 12 or 24 items. All used 4 different colors picked from the total set of 8. Of these items exactly half could contain the target (‘relevant’ items), and this half of the items had either 1, 2 or 3 different colors, assigned to an equal number of items (see Table 1 for details). The remaining colors were also each assigned to an equal number of the remaining items (‘irrelevant’ items). Positive cues could refer to either one (“the target is of color …”), two, or three potential target colors (“the target is on one of the following colors: …”). Similarly, negative cues could refer to either one, two or three colors that could not contain the target. Consequently, if all cues are used to create a “template for inclusion,” the 1-color positive cue would be most comparable to the 3-color negative cue and vice versa, and the 2-color negative and positive cues should also be comparable. In addition, a neutral condition was included, in which the target could appear on any of 4 colors, which were present on an equal number of items. Positive, negative as well as neutral cue trials were blocked. There was a total of 7 different cue conditions (1-positive, 2-positive, 3-positive, 1-negative, 2-negative, 3-negative, neutral; Table 1). Each cue condition was repeated 6 times, resulting in a total of 42 (7x6) blocks, of 8 trials each, resulting in a total of 336 trials. Each block consisted of 4 trials with a 12-item array and 4 with a 24-item array, and each array size had 2 target-present trials and 2 target-absent trials. Order of trials in a block was random. Fourteen volunteers (age 26±3 years, 8 female) participated. Eye movements were recorded with an Eyelink-1000 (SR Research) eye-tracking device at 1000Hz with default settings for saccade and fixation detection. Fixations were attributed to objects by the same procedure described in experiment 1, thereby 97% of fixations observed in experiment 2 were attributed to a specific item. An EIZO FlexScan F77S controlled by a Windows-PC at a distance of 73cm was used for presentation. In all other respects, experiment 2 was identical to experiment 1.

**Table 1:** Conditions in experiment 2.

		Items of Color				
Condition	Cue, the target will be:	1	2	3	4	Relevant colors
1-positive	Color 1	***6***	2	2	2	1
2-positive	Color 1 or 2	***3***	***3***	3	3	2
3-positive	Color 1, 2 or 3	***2***	***2***	***2***	6	3
1-negative	Not color 1	6	***2***	***2***	***2***	3
2-negative	Not color 1 and 2	3	3	***3***	***3***	2
3-negative	Not color 1,2, and 3	2	2	2	***6***	1
neutral	The target can be any color	***3***	***3***	***3***	***3***	4

There are 7 different cue-conditions, three different positive and negative cues, as well as one neutral cue. Listed are the cues given to participants, with how many items out of twelve are of each color, and how many relevant colors there are in the array. The items that are potential targets are in bold/italic face. Analyses is done using the number of relevant colors, as this assesses any color switching costs directly. For 24-items trials all item numbers need to be doubled.

## Results

### Experiment 1

#### Search accuracy

**Fig 3:**
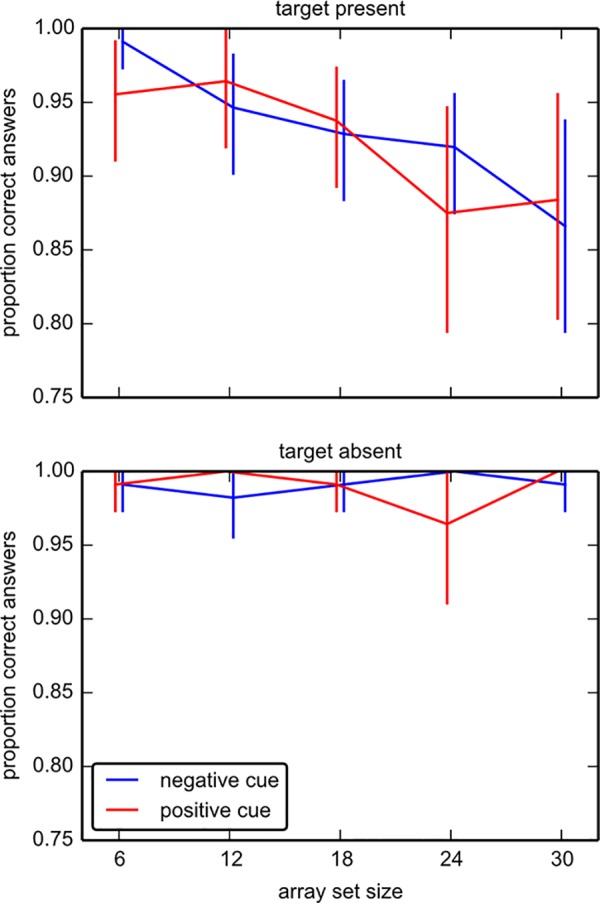
Correct responses in experiment 1. Target-present trials (top) and target-absent trials (bottom) in both cue types; positive (blue) and negative (red). Bars show (bootstrap) confidence intervals.

Our analysis of search accuracy indicates that participants could find the targets regardless of the cue type. We found no difference in accuracy following positive or negative cues. General mixed-effects models were calculated for the proportion of correct responses (search accuracy, Fig 3) in both cue types. In the target present case, *array set size* had an effect (Wald test χ^2^(4) = 16.86, p = .002), whereas *cue type* had no effect on accuracy (Wald test χ^2^(1) = 0.10, p = .75), nor did the interaction *cue type* x *array set size* (Wald test χ^2^(4) = 4.03, p = 0.40). When the array set size increased, participants missed a present target more often. In the target absent case, neither *array set size* (Wald test χ^2^(4) = 3.06, p = .55) nor *cue type* (Wald test χ^2^(1) = 0.0, p = .99) had an effect on accuracy. There was no interaction (Wald test χ^2^(4) = 0.06, p = 0.99).

We analyzed search performance by calculating d’ and c scores separately for each participant and cue type. For negative cues, the individuals’ sensitivity indices (d’) were on average slightly larger (mean: 3.72, ranging from 2.80 to 4.48) than for positive cues (mean: 3.70 ranging from 2.12 to 4.48), but this difference was not significant (t(13) = 0.162; p = .874). Similarly, we did not find that the (c) for negative cues (mean: 0.28, ranging from -0.16 to 0.55) and the criterion for positive cues (mean: 0.28, ranging from 0.00 to 0.69) were different (t(13) = 0.009; p = .993). Hence performance was close to ceiling and highly similar for the two cue types, as was the response criterion.

#### Response times

**Fig 4:**
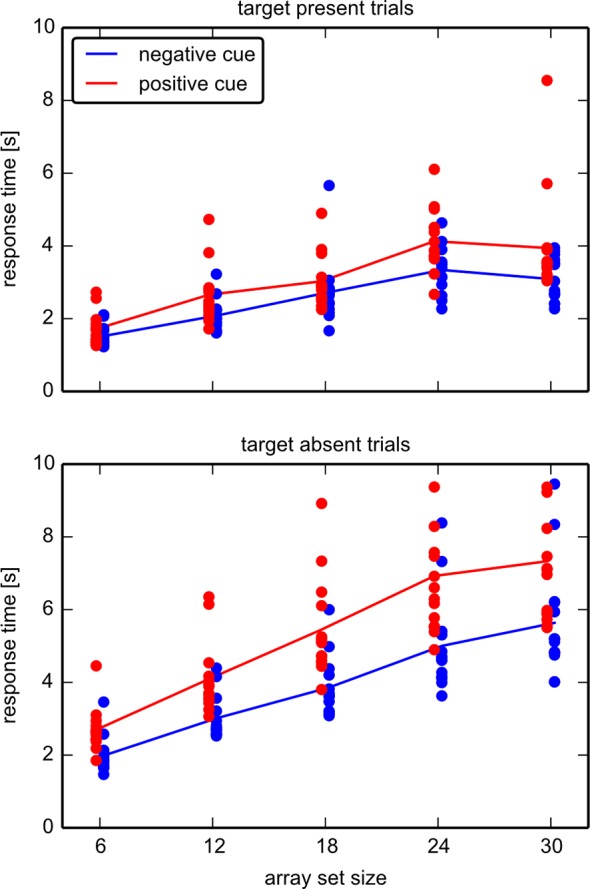
Response Times in experiment 1. Shown are the response times in target present trials (top) and target absent trials (bottom) in both cue types; positive (blue) and negative (red). Dots denote mean values for correct answers of each individual and number of items, lines the means for each group. The slopes of linear fits were determined for target absent trials for set sizes 6–24, where linearity seems to be satisfied. All participants exhibited a higher slope in “negative cue” (mean value 236ms/item) versus “positive cue” (mean value 158ms/item) condition.

In both the target-absent and the target-present conditions, positive cues resulted in shorter response times as compared to negative cues. The response time increased with array set size. This indicates that participants indeed engaged in a serial-type search, where response time is dependent on the array set size. The response times (Fig 4) were analyzed with separate repeated measures ANOVAs for target absent and present. For target absent, the two-way ANOVA yielded significant main effects for *cue type* (F(1,13) = 128.7, p < .001), and *array set size* (F(4,52) = 119.6, p < .001), and a significant interaction between *array set size* and *cue type* (F(4,52) = 13.4, p < .001). For target present, the two-way ANOVA yielded significant main effects for *cue type* (F(1,13) = 21.2, p < .001), and *array set size* (F(4,52) = 64.8, p < .001). The interaction between *array set size* and *cue type* was not significant after Greenhouse-Geisser correction (F(4,52) = 1.9, p = .18).

#### Fixations

**Fig 5:**
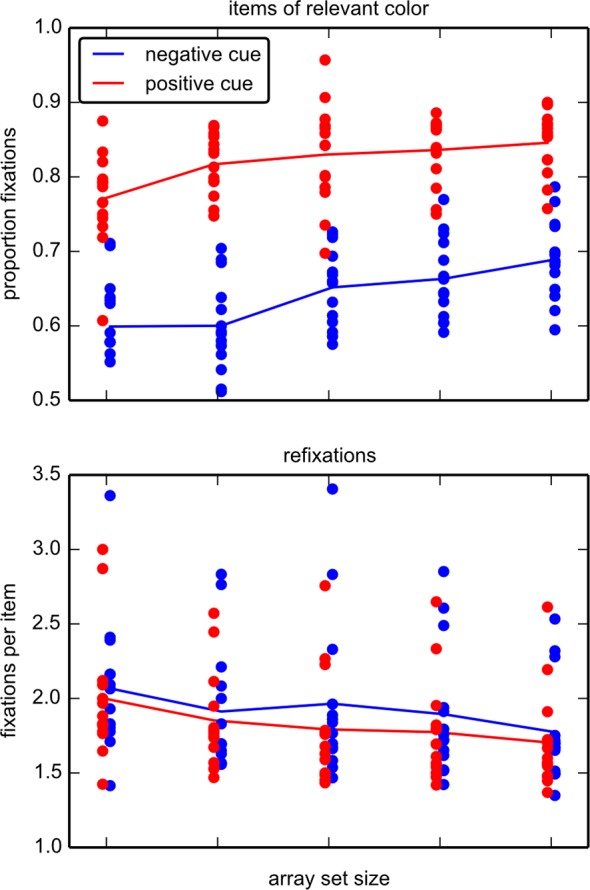
Fixations in experiment 1. (Top) The proportion of fixations on the relevant half of items is shown (only target absent trials). In positive cue (blue), relevant items are of the color given in the instruction. These items are spatially identical in the negative cue (red), but of various colors. Dots depict mean values of each individual, lines are the means for each group. As half of the items are relevant and half of the items irrelevant, 0.5 represents chance level. (Bottom) Number of fixations per fixated item. Items are refixated significantly more often in negative cue (red) compared to positive cue (blue), despite high individual variation.

Both positive and negative cues were used to guide gaze toward relevant locations, although positive cues lead to more efficient guidance of gaze. This is reflected by the proportion of fixations landing on relevant locations (only fixations that were attributed to items are taken into account), which was above chance level for both positive (t(13) = 32.9; p < .001) and negative cues (t(13) = 11.4; p < .001). However, the proportion of fixations on relevant locations was higher in positive-cue blocks than in negative-cue blocks (Fig 5A), although the number of relevant locations was the same across conditions. This was confirmed by a main effect for *cue type* (F(1,13) = 225.2, p < .001) as well as for *array set size* (F(4,52) = 18.1, p < .001), with no interaction (F(4,52) = 2.02, p = .105, repeated measures ANOVA).

For each participant, we correlated response time in correct target-present trials to the time needed to first fixate the target. We found correlations in the range from r = 0.75 to r = 0.99 (average r = 0.92). Degrees of freedom varied between 51 and 78, since incorrect trials were excluded. All correlations were significantly different from 0 at p < .0001: well below the Bonferoni corrected 5% level (0.05/14 = 0.0036). This indicates that the time needed to first fixate the target was the main determinant of response time.

We hypothesized that the decision on which item to fixate next affects the duration of the current fixation and that this decision takes longer when using a negative cue as compared to a positive cue. Therefore we also compared fixation durations between positive and negative cues. Fixation times with a negative cue were indeed longer than with a positive cue (group mean in fixations on relevant items: 203 ms vs. 193ms; in fixations on irrelevant items: 181ms vs. 170ms). There were significant main effects for *cue type* (F(1,13) = 26.3, p < .001) and *item relevance* (F(1,13) = 57.3, p < .001), with no interaction (F(1,13) = 0.21, p = 0.65, repeated measures ANOVA). In other words, fixations with a negative cue were about 11ms longer than fixations with a positive cue, and this could signify that the process of deciding where to fixate next took longer when using a negative cue for the decision. Furthermore, fixations on relevant items were 22ms longer than fixations on irrelevant items. This implies that participants try to discern whether or not the currently fixated item is the target more frequently when they are fixating a relevant item.

Another measure of gaze guidance was how often items are refixated. If search followed an optimal strategy, no item would be fixated more than once. However, there was a substantial number of refixations for each cue type (Fig 5B). The analysis of the number of fixations per fixated item showed main effects for *cue type* (F(1,13) = 10.5, p = .006) and *array set size* (F(4,52) = 11.36, p < .001) and again no interaction (F(4,52) = 0.92, p = 0.46): negative cues led to more refixations than positive cues and refixations decreased with set size.

**Fig 6:**
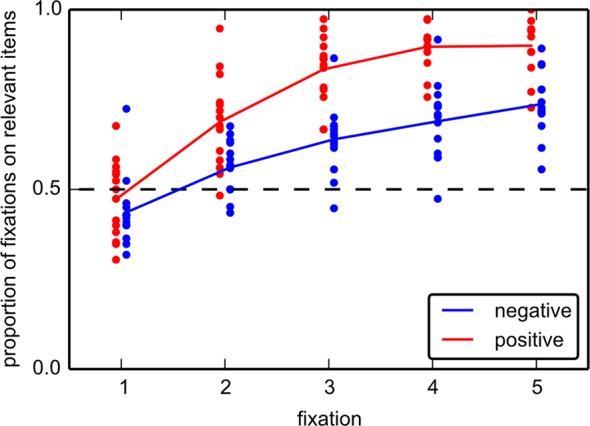
Proportion of fixations on relevant items for the first five fixations in each trial for experiment 1. Only fixations starting after the onset of the array are used. At the start of the trial, there is a small, but significant difference between positive and negative cues. The first fixation following a negative cue is even guided toward irrelevant items. A positive cue guides participants towards relevant items immediately, but negative cues also provide correct guidance from the second fixation on.

Finally, we analyzed how guidance of gaze developed from fixation to fixation (Fig 6). We performed a two-way, repeated measures ANOVA on the proportion of fixations on relevant items, with *cue type* (positive vs. negative) and *fixation number* (1.5) as factors. The proportion of fixations on relevant items following positive cues is higher than in negative cues (Fig 6), which is confirmed by a main effect of *cue type* (F(1,13) = 97.22, p < .001). Guidance of gaze also improves with successive fixations within a search (Fig 6), which is confirmed by a main effect of *fixation number* (F(4,52) = 95.57, p < .001). Finally, the rate of increase was different between positive and negative cues, which is demonstrated by an interaction between *cue type* and *fixation number* (F(4,52) = 6.26, p < .001). On the first fixation after stimulus onset, there seems to be little benefit from either cue type. The proportion of first fixations that fall on relevant items (47.2%) following a positive cue did not show a significant deviation from chance level (t(13) = 0.96, p = .353, t-test against 50%). Negative cues first even guided attention incorrectly, with a proportion of fixations on relevant items of 43.6%, which is below the 50% chance level (t(13) = 2.47, p = .03). For the second fixation both positive cues (t(13) = 5.48, p < .001) and negative cues (t(13) = 3.04, p = .009) showed above chance guidance, and guidance kept developing over the first 5 fixations. However, the guidance provided by negative cues developed slower than that provided by positive cues (Fig 6).

### Experiment 2

To disentangle possible effects of color switching costs, experiment 2 used various numbers of cued colors for both negative and positive cues.

To assess performance, we calculated d’ and c for each participant in positive and negative cues. We found that d’ was slightly lower for negative (mean: 3.76, ranging from 2.31 to 4.40) than for positive cues (mean: 3.94, ranging from 3.22 to 4.30), but this was not significant (t(13) = 1.44; p = .172). Similarly, c was slightly higher (more liberal) for negative cues (mean: 0.437, ranging from -0.129 to 0.892) than for positive cues (mean: 0.375, ranging from -0.142 to 0.847), but again this difference is not significant (t(13) = 0.908; p = .381). We also compared the overall d’ and c scores in experiment 1 with those in experiment 2. Neither d’ (t(26) = 0.409; p = .686) nor c (t(26) = 1.55; p = .133) differed between the experiments, so that performance in experiment 2 is also close to maximum and not distinct from performance in experiment 1. Only the correct trials (94.8% of all trials in experiment 2) were used for analyses of response times and fixations.

**Fig 7:**
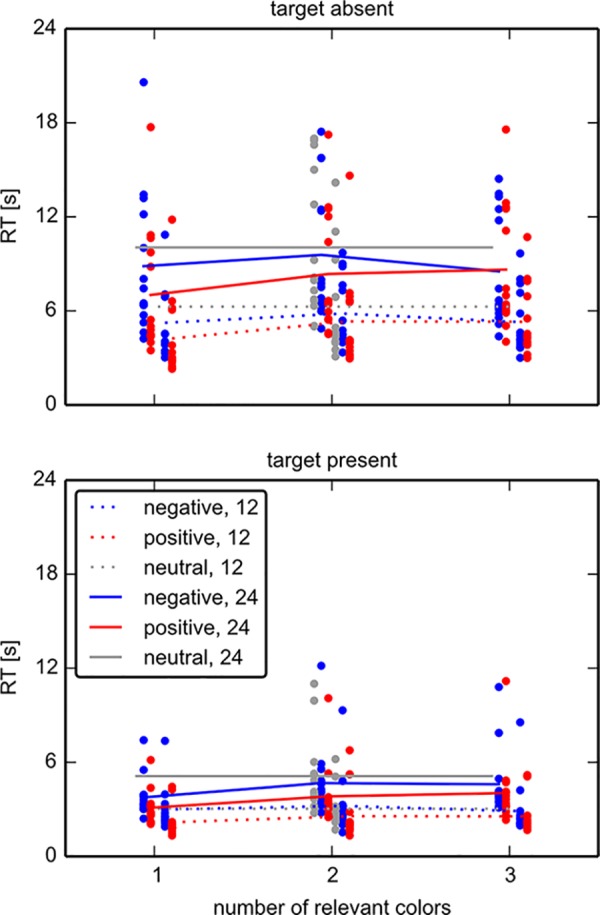
Response times in experiment 2. The response times for target absent trials (top) and target present trials (bottom) are shown, split by cue type and array size. The main determinant of response time is array size, but cue type also plays a consistent role with positive cues eliciting the fastest responses, and neutral cues the slowest responses.

We found that both positive cues and negative cues yielded faster responses than neutral cues (Fig 7; positive vs. neutral: t(13) = 5.17, p < .001; negative vs. neutral: t(13) = 2.39, p = .017). Direct comparison between positive and negative cues showed a behavioral advantage for positive cues (t(13) = 8.54, p < .001). We found no evidence that repeatedly cuing relevant colors throughout a block of 8 trials incurs a different benefit on RT than repeatedly cuing irrelevant colors (t(13) = 0.653, p = .525). Overall, the beneficial effects of positive and negative cues over neutral cues, and the larger benefits for positive cues, confirm the eye-tracking data of experiment 1.

**Fig 8:**
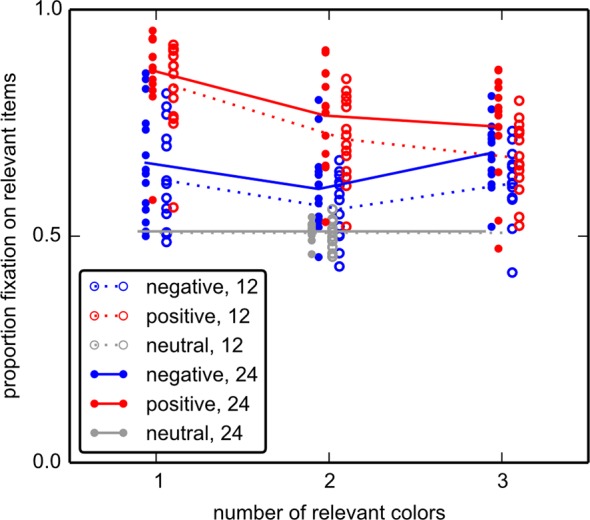
Proportion of fixations on relevant items in experiment 2. The proportion of fixations on relevant items for each cue type and array size are plotted over the number of relevant colors in the array. Cue type has the largest effect on guidance of gaze towards relevant items, with positive cues being more effective than negative cues, which in turn are more effective than neutral cues. Array size modulates this, so that with larger arrays, gaze is guided towards relevant items more efficiently.

A similar pattern was observed for the proportion of fixations on relevant items (see Fig 8). For negative cues, 62.5%±8.2% (mean ± standard deviation) of fixations fell on relevant items, while for positive cues this proportion was 76.5%±8.7%. Both numbers are above chance (50%, since in all conditions half of the items are relevant; t(13) = 5.50, p < .001, and t(13) = 11.01, p < .001, respectively). Direct comparison again shows a significant benefit of the positive cues over negative cues (t(13) = 9.398, p < .001), confirming the behavioral observations of experiment 1.

In short, experiment 2 replicates the difference between negative and positive cues demonstrated in experiment 1 across different numbers of relevant colors in the array, and shows a benefit of negative cues over neutral cues.

In experiment 1, a negative cue (always of 1 irrelevant color) had been associated with three relevant colors, while a positive cue directly indicated a single relevant color. If either cue type had been used to generate a “template for inclusion” this would mean that the cost of switching between the three relevant colors following negative cues–and not the framing of the cues–might explain the effects found in experiment 1. Experiment 2 allowed us to dissociate between these factors. We performed two repeated-measures ANOVAs, one on response times, and one on the proportion of fixations on relevant items. Both models included *array size* (12 or 24) and the predictor of interest, *cue type* (positive or negative) as a factor. To control for color-switching costs the *number of relevant colors* (1, 2 or 3) was included as within-subject covariate. If color switching does explain the effects found in experiment 1, then there should be no effect of *cue type* in these ANOVAs. Since the neutral condition does not distinguish relevant from irrelevant items, it was not included in this analysis. For the ANOVA on RT, we do find an effect of *cue type* (F(1,13) = 73.0; p < .001) as well as of *array size* (F(1,13) = 68.93; p < .001), and an interaction (F(1,13) = 10.51;p = .006). For the proportion of fixations on relevant items, we also find an effect of *cue type* (F(1,13) = 88.33; p < .001) as well as of *array size* (F(1,13) = 60.86; p < .001), but no interaction (F(1,13) = 0.037; p = .850). This shows that controlling for the number of relevant colors in the array cannot explain away the difference between the effects of positively and negatively framed cues.

**Fig 9:**
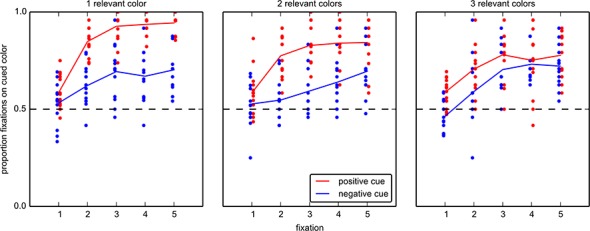
Proportion of fixations on relevant items for the first five fixations in each trial in experiment 2. Left: relevant items are of one color, middle: relevant items are of two colors, right: relevant items are of three colors. The same general pattern as seen in Fig 6 can be observed for each number of relevant colors in the array. The first fixation following a negative cue of one irrelevant color (leaving three relevant colors) might be guided towards this color–it is the only average that is below chance level–but this is not significant. Positive cues immediately guide participants’ gaze toward relevant items. Negative cues also provide correct guidance, but this develops more slowly than for positive cues.

For experiment 2, we also investigated the time course of guidance by positive and negative cues (Fig 9). We performed a repeated measures ANOVA on the proportion of fixations on relevant items with the factors *cue type* (positive vs. negative), *fixation number* (1.5) and the *number of relevant colors* (1, 2 or 3). There is a main effect of *cue type* (F(1,13) = 134.9, p < .001), showing that positive cues elicit a higher proportion of fixations on relevant items. There is also a main effect of *fixation number* (F(4,52) = 50.71, p < .001), which shows that the proportion of fixations on relevant items increases with time. There is an interaction between *cue type* and *fixation number* (F(4,52) = 5.203, p = .001), which shows that the rate of increase of the proportion of fixation on relevant items over time, is different for the two cue types. Furthermore, there is a main effect of the *number of relevant colors* (F(2,16) = 13.90, p < .001), and an interaction between *cue type* and *number of relevant colors* (F(2,26) = 11.51, p < .001), but not between *number of relevant colors* and *fixation number* (F(8,104) = 1.564, p = .198). There is also a three way interaction between all three factors (F(8,104) = 3.41, p = .002), which indicates that the difference in the rate of increase between the cue types, changes with the number of relevant colors. The only average proportion of fixations that is below 50% is that for the first fixation following a negative cue for 3 relevant items (46.4%), but–unlike for experiment 1 –this fails to reach significance (t(13) = 1.676 p = .12). Aside from this, we observe the same patterns as in experiment 1: negative cues guide gaze towards relevant items, but this develops slower than guidance by positive cues.

## Discussion

We investigated the efficiency of negative vs. positive cue types in visual search. Our results show that negative cues can guide search, but are considerably less efficient than positive cues.

All eye-movement measures tested, i.e., proportion of fixations on relevant locations, fixation duration and refixation rate, show a benefit for positive cues for guiding gaze in search, and thus may contribute to the reaction time advantage when given a positive cue compared to a negative cue [17]. These observations suggest that search guidance makes more efficient use of positively framed information.

Previous studies have already revealed some indications that search might benefit from negative cues. The content of working memory can flexibly both inhibit and facilitate attention, as the task demands [15]. Similarly, repeating distractors across trials helps to ignore them during search [16], also hinting at a benefit of spatial information on what not to look for [19]. A recent study examined a search task with two differently colored hemifields of items, giving either the targets color or the non-target color, and showed visual search benefited not only from the positive color cue, but also, to a lesser extent, from the negative cue type, compared to providing no information [17]. However, in that study color and spatial information were correlated. Thus it is not clear whether negative color information alone is beneficial for visual search [18] [20].

In our paradigm, we eliminated spatial cues by randomly distributing targets and non-target items across the search arrays. We designed our task such that search had to be performed by foveating each potential target item (i.e., each “relevant” location) individually. The decline of performance with set size confirms that this kind of search is indeed inefficient and most likely serial [1] [2]. In the absence of a neutral condition (as in our experiment 1), evaluating performance data alone would not be able to tell whether the negative cues are used at all. However, the number of fixations of relevant items compared to irrelevant ones is significantly higher than chance level, which shows that the information contained in the negative cues is both available and used. The comparison to the neutral condition in experiment 2 also confirms this finding for performance and response time measurements.

Eye-tracking data reveal pronounced differences for positively vs. negatively guided search. Perfect guidance would imply that only the relevant half of locations is included in the serial inspection. In experiment 1 the positive cue type elicits a fraction of fixations on items of the relevant color that usually exceeds 80% (Fig 5), in conformance with previous data [29]. In contrast, with a negative cue the proportion of fixations on relevant items drops to about 60%-65%, depending on array size. Even if this difference may appear small, it implies that about a third more items are inspected before a decision is reached. Together with ~6% longer fixation durations and ~8% increased number of refixations, the disadvantages of negative cues aggregate to the approximately 50% response time difference between negative and positive cues, which we observe here. As such, the eye-movement analysis therefore allows to break down the response time differences into its components. The most important component appears to be indeed (mis-)guidance to irrelevant locations. Experiment 2 confirms this finding, as across the conditions a positive cue leads to a higher proportion of fixations on relevant items.

On the basis of the present results, we might speculate why guidance under positive cues is more efficient than guidance under negative cues. First, there might be an acquired tendency to perform search using positively framed information. This tendency appears to be reasonable considering the fact that visual working memory is strongly limited [30] [31], and defining an object by the absence of feature values typically requires more capacity (“a strawberry is not yellow, not blue, not …”) than defining it by the presence of feature values (“a strawberry is red”). Second, the longer fixation times indicate that negative cueing might require an additional intermediate inferential processing step, i.e., the translation of the negative cue into a positive one [32]. This step might occur either only once at the beginning of the search task, or repeatedly during the search process. However, since controlling for the number of relevant colors does not eliminate the effect of cue type, it is likely that a “template for rejection” is used instead.

Guidance to irrelevant locations occurs more often on the first fixation following a negative cue in experiment 1, where the proportion of fixations on relevant items is even below chance. In experiment 2, a similar, but non-significant tendency is present, but only for the case of 3 relevant colors. This confirms previous behavioral findings from using negative color cues [33]. With a short stimulus onset asynchrony (SOA), performance suffered when the color of a distractor among four items was cued (as opposed to the location [19]), but this surprising effect disappeared with a longer SOA. Analogously, we find that with prolonged search (i.e., on later fixations, or with larger arrays), the benefit of both positive and negative cues increases throughout a trial to an extent that for both cue types overall efficiency is increased in larger arrays. In sum, our data show that both positive and negative cues improve search. This manifests itself in reaction times and fixations on relevant items. Positive cues are more effective than negative cues, but both become more effective over the course of the search.

In many visual search experiments color has been identified as the most effective cue [34] [35] [36]. Thus, color is a reasonable choice for the initial investigation of eye movements during search when given negative cues. Whether or not our results on negative cues extends to other features is as of now speculation, but it is well conceivable that features that show weaker effects in positive search (e.g., size, shape) are also less effective as negative cues.

## Conclusion

Here we investigate how gaze guidance following negative cues is different from guidance following positive cues in search. We find that color-only negative cues can be used to guide gaze and thus to accelerate search. However, negative cues are less effective than positive cues in directing visual attention to relevant items, and dwell time per item is longer, thus search is less efficient. For both cue types, their effectiveness, as measured by the number of fixations on relevant items, increases over the course of the search.
